# Experiences in managing problematic crystal methamphetamine use and associated depression in gay men and HIV positive men: in-depth interviews with general practitioners in Sydney, Australia

**DOI:** 10.1186/1471-2296-9-45

**Published:** 2008-08-15

**Authors:** Deborah C Saltman, Christy E Newman, Limin Mao, Susan C Kippax, Michael R Kidd

**Affiliations:** 1 Institute of Postgraduate Medicine, Brighton and Sussex Medical School, Brighton, UK; 2 National Centre in HIV Social Research, The University of New South Wales, Sydney, Australia; 3 Discipline of General Practice, The University of Sydney, Sydney, Australia

## Abstract

**Background:**

This paper describes the experiences of Australian general practitioners (GPs) in managing problematic crystal methamphetamine (crystal meth) use among two groups of male patients: gay men and HIV positive men.

**Methods:**

Semi-structured qualitative interviews with GPs with HIV medication prescribing rights were conducted in Sydney, Adelaide and a rural-coastal town in New South Wales between August and October 2006. Participants were recruited from practices with high caseloads of gay and HIV positive men.

**Results:**

Sixteen GPs were recruited from seven practices to take part in interviews. Participants included 14 male GPs and two female GPs, and the number of years each had been working in HIV medicine ranged from two to 24. Eleven of the GPs who were based in Sydney raised the issue of problematic crystal meth use in these two patient populations. Five key themes were identified: an increasing problem; associations with depression; treatment challenges; health services and health care; workforce issues.

**Conclusion:**

Despite study limitations, key implications can be identified. Health practitioners may benefit from broadening their understandings of how to anticipate and respond to problematic levels of crystal meth use in their patients. Early intervention can mitigate the impact of crystal meth use on co-morbid mental illness and other health issues. Management of the complex relationships between drug use, depression, sexuality and HIV can be addressed following a 'stepped care' approach. General practice guidelines for the management of crystal meth use problems should address specific issues associated with gay men and HIV positive men. GPs and other health practitioners must appreciate drug use as a social practice in order to build trust with gay men to encourage full disclosure of drug use. Education programs should train health practitioners in these issues, and increased resourcing provided to support the often difficult task of caring for people who use crystal meth. Greater resourcing of acute care and referral services can shift the burden away from primary care and community services. Further investigation should consider whether these findings are reproducible in other general practice settings, the relationship between depression, drug use and HIV medication, and challenges facing the HIV general practice workforce in Australia.

## Background

The chemist and pharmacologist Fritz Hauschild developed the sympathomimetic agent Pervitin (metamphetamin) in the 1930s [1]. Methamphetamine, a derivative of amphetamine, was widely prescribed in the 1950s and 1960s as a medication for depression and obesity, reaching a peak of 31 million prescriptions in the United States in 1967 [2]. Now widely available in many nations as a street drug in either base, powder or crystalline ('crystal meth' or 'ice') forms, this central nervous system stimulant can be injected or ingested nasally or orally. The most frequently reported positive effects are feeling more awake or alert, increased energy and sociability, and aphrodisiac qualities, while negative effects reported by users include 'comedown', paranoia and inability to sleep [3]. A study of regular methamphetamine users in Sydney found that most preferred base and ice because they 'provided a more intense and longer lasting high' [4].

In a poignant reversal of the original application of methamphetamine as an antidepressant treatment, rates of depression diagnosis and prescription of antidepressants are significantly higher among Sydney methamphetamine users than the general population [4]. While there is little evidence that depressed people are more likely to use crystal meth [5], dependence on and recent use of crystal meth has been associated with reported symptoms of depression in multiple studies [eg. [6]]. Methamphetamine withdrawal symptoms, including fatigue, anhedonia, depressed mood, paranoia and hypersomnia, are common to depression and are often severe enough to precipitate relapse [5]. These effects are reported to be more severe with crystal meth than other forms of methamphetamine [4].

The behavioural data indicate that crystal meth is becoming an increasingly normative part of the Australian gay scene, associated mostly with dance parties but also with sexual activity in other settings for some users [3]. Importantly, the research indicates that most of the gay men who use crystal meth regularly manage this use with few problems [7]. However, there is still a considerable minority of men who report dependent levels of crystal meth use [7]. Just over 20% of gay men participating in the 2007 Sydney Gay Community Periodic Survey reported using crystal meth in the previous six months [8]. This had increased significantly from around 12% in 2002. Amongst those who used crystal meth in the six months prior to the 2007 survey, nearly half used it at least monthly [8]. In people living with HIV/AIDS (of which the great majority in Australia are gay men [9]), depression has been shown to be associated with a history of illicit drug use [10], and other studies have suggested that co-morbid depression and drug use are associated with decreased HIV treatment uptake, treatment adherence problems and virological suppression [11]. In sum, this literature suggests that while some gay men and some HIV positive men may be able to integrate occasional crystal meth use into their lives with minimal impact, others are using to a degree that can have problematic consequences for health as well as social and economic wellbeing.

This paper explores how reported instances of problematic crystal meth use among gay men and HIV positive men are affecting the work of general practitioners (GPs) in Australia. Although there has been growing concern in gay communities about rates of crystal meth use [12], we believe this to be the first analysis of the experiences of Australian GPs who work with gay men and HIV positive men who use crystal meth.

## Methods

The data reported in this paper are drawn from the Primary Health Care Project on HIV and Depression, an interdisciplinary and multi-method study exploring the complex relationships between sexuality, HIV and depression. Written informed consent was obtained from all participants. Ethics approval was granted by the National Research and Evaluation Ethics Committee of the Royal Australian College of General Practitioners (Approval No: RACGP NREEC 05/35) and the Human Research Ethics Committees of The University of New South Wales and The University of Adelaide.

The study methodology has been described elsewhere [13,14], but there are several aspects that are important to report in relation to this paper. First, this paper is focused on data gathered in the first of three stages of the project. This stage comprised semi-structured interviews with s100 HIV prescriber GPs, eg. primary care physicians trained and accredited to prescribe HIV medication. Sixteen interviews were conducted with GPs (14 male, 2 female) who were based in three Australian cities: two state capitals, one large (Sydney) and one small (Adelaide); and one rural-coastal city. The seven practices in which the GPs were based each treated a high caseload of the gay or homosexually active men and HIV positive men that lived in those areas.

The second important aspect of the method is that this paper is focused on the experiences reported by the eleven GPs who were based in Sydney, each of whom raised the issue of problematic patient crystal meth use during the interviews. A semi-structured interview format was employed in the interviews to allow for consistency as well as flexibility in remaining responsive to the range of issues and ideas that can emerge in an open-ended conversation. Following the aims of the study, GPs were asked to focus on their experiences as they related to the treatment of gay men and HIV positive men. However, there was minimal guidance in terms of what the GPs may or may not find most topical or challenging about those experiences. A thematic analysis of deidentified transcripts was applied using NVivo 7 [15] and following the principles of open and axial coding [16]. Reliability was established through a process of independent coding and discussion amongst the research team. This process identified key themes on: how gender and sexuality differentially shaped the experience of depression in gay men in comparison with heterosexual men [13]; how a long term and trusting relationship between doctor and patient increased the likelihood of a timely diagnosis of depression in HIV positive men [17]; and how the growing range of clinical and contextual challenges for GPs working in HIV affected their capacity to manage depressive illness in both gay men and in HIV positive men.

The issue of patient crystal meth use was only raised in the eleven interviews conducted with Sydney-based GPs. These eleven GPs represent more than one third of all of the s100 HIV prescriber GPs working in the inner suburbs of Sydney [18]. The rest of this paper will explore five key themes in the reported experiences of these eleven GPs, focusing on their observations of problematic crystal meth use in gay men and in HIV positive men in Sydney. It is important to note that GPs often spoke about these two patient populations interchangeably. Where a distinction was clear this has been reported as such, however, the more common conflation or substitution of their identifying features is discussed in the final section of the paper.

## Results

### Increasing problem

The first and most common theme in the reported experiences of GPs is that crystal meth use has dramatically increased in recent years among the populations of gay men and HIV positive men they treat in Sydney:

I mean recreational drugs have always been around, but not to the extent where people are not complying with their medication so much and getting depressed, and getting physically and mentally ill with things like crystal meth ... I mean I've been away and then this last year this seems to be an ongoing problem that people are ... trying to deal with. [GP3]

An important characteristic of this theme is that not only is crystal meth use growing, but that problematic levels of crystal meth use are being observed in gay men and positive men who do not have a history of problematic levels of drug use:

I'm really surprised at the level of crystal use out there.... And the folk who I would least expect to actually access it, because it's so freely available, and part of the party scene now, who are now accessing and who are now using. And have been actually now shall we say 'hooked in', and are now becoming regular users. And I've been quite surprised at who's actually taking this up. Now these are folk that I would assume would have some degree of control over their actions... I'm talking about men in their thirties and forties who have been around, who know the scene, but who are now actively involved in crystal use ... I'm, it really worries me the extent of addiction that's actually happening, about people I would least expect. [GP7]

### Associations with depression

A second theme focuses on the observed associations between problematic levels of crystal meth use and depression in both gay men and in HIV positive men:

In terms of depression at the moment, one of our major problems in this environment at the moment would be the crystal 'epidemic', or whatever you want to call it. And that can make a lot of people very depressed! [GP4]

But I would say, amphetamine use is pretty high and linked with this depression, particularly linked with depression. [GP10]

However, this association is not seen as simple or straightforward; rather, the associations between depression and crystal meth use are represented as quite complex and difficult to tease apart:

I mean most boys using large amounts of crystal are depressed. And how you address that in the context of perhaps ongoing crystal use and work out what's depression as such or what's a drug effect, or why someone's using crystal, I think that's a major problem we're dealing with at the moment. [GP4]

Well, why are people [having] drug and alcohol issues in the first place? Is it because there is a pre existing depression and this is actually part of trying to mask that? It's drawing a long bow to say that will, going [down] that path, cause a depression. Is there a pre existing depression here anyway? [GP7]

### Treatment challenges

A third theme describes the various ways in which crystal meth use creates treatment challenges for GPs, including in the effective clinical management of depression:

If someone's injecting a lot of amphetamines, it's very hard to diagnose depression. Also medication's probably not going to be particularly useful. [GP6]

Post party depression. It's very common, and then a few days later they're fine. When people are withdrawing from substances, I've seen people get diagnosed, put on major, major tranquillisers and antidepressants. And I don't, I'm not sure if they really needed it. [GP9]

There's a lot of substance abuse that negates antidepressant therapy, things like that. I mean, [antidepressant drug] isn't going to do much good if people are doing crystal meth every weekend, do you know what I mean? ... And the patients don't necessarily tell you that they have these problems, or that they are using these things. It's very common to see. Gay men come in here and say, "I'm depressed and I really want to do something about it. I've heard about antidepressant medication. I want to go on it." And they don't tell you, even if you ask them specifically, that they are using stimulants recreationally, until some time later. Yeah, that's a big obstacle. [GP5]

Treatment challenges are further complicated when considering the clinical management of HIV alongside co-morbid depression and drug use:

However, with the advent of our HIV population, with the use of, particularly the use of ice, particularly the use of crystal... I have actually seen stable people who I thought were quite well controlled on their, either their cognitive behavioural therapy or their antidepressant medication, talk to me about suicidal intent coming down off their crystal. And that's creating some problems for me at the moment. [GP7]

Positive men and depression? No, it's this whole sort of soup of depression, self esteem, drug use. Trying to tease them out and treat. So I think you really need to make sure you're treating all of them. [GP11]

### Health services and health care

A fourth theme focuses on the capacity of health services to provide effective care for gay men and HIV positive men who are experiencing problematic levels of crystal meth use, particularly in terms of their mental health care needs:

There needs to really urgently, urgently address the drug issue. People who become acutely depressed or psychotic or both, under the influence of drugs. And rehabilitation for it. Because generally they're just sent out of hospital. They ring you up and go, "They've just been discharged. Filled them up with tranquillisers and discharged them on Monday morning". And there's no follow up, there's nothing ... And then the person doesn't come back, like I can't get hold of them. So I don't, I think there has to be a better system in place so that these people don't cause social harm by going berserk in the community as well ... And to get someone into a private psychiatrist interested in drug and alcohol can take months. And that, even costlier are those private rehab places. [GP9]

Related to health service capacity are the attitudes of health practitioners, including the observed reluctance of some referral specialists to accept clients who use crystal meth:

And I won't name names, but I send to, a lot of these people, to a certain psychiatrist. And he said, "Don't send me any more!" [laughs] Because he's fed up with them. And I would be too, I guess. [GP9]

GPs commonly stressed the importance of providing non-judgmental support to men who were using crystal meth, whether or not they felt it was a problematic behaviour. This aspect of the theme implies that effective health care requires the absence of both social prejudice and criminal implications:

Because most people would come to us for a sexual health reason in the beginning and like the atmosphere or like the doctors, or whatever. Most of them liked the non judgemental ... And being in the city, they liked being able to say that they were feeling a bit tired but could it have been the five trips and crystal they had on the weekend. And we didn't ring the police and fall off our chairs. So they'd come back with an earache. [GP8]

### Workforce issues

The final theme describes some key issues facing the GP workforce members who provide care to these populations of gay men and HIV positive men in inner city Sydney. This includes GPs feeling unprepared to support the number of men seeking treatment for crystal meth addiction and associated co-morbidities:

And I'm seeing people I'd least expect saying to me, "I can't stand this. I've just got to get off this. How do I do it? [GP7]

There's a problem with crystal meth usage in Sydney, all right? And dealing with addiction and substance abuse, I feel less comfortable with. I don't feel I have the expertise to deal with that, so I tend to send them that way for that. [GP11]

An important aspect of this theme is the potential for burnout in those health care workers – particularly GPs who are HIV medication prescribers – who are responding to problematic levels of crystal meth use on top of a growing range of other contextual stressors:

I think it's nasty at the moment, and I think it's nastier for the health worker too. Especially with crystal meth ... people are nasty, they're horrible and aggressive. And you become it too, I think. Because that's the only way you can, because you've got to make very strong borders, boundaries for them to not cross. And people become more unreliable. They're narkier, they have nasty withdrawals. And I think it's a big issue. And I think it's also a very big issue with a lot of, like people working in HIV where there may be... A lot of people are saying there's no evidence, but there's a lot of anecdotal talk ... And that a lot of people are going to burn out. And as you know there are fewer and fewer doctors wanting to do it because they've been told that it's really hard to do HIV. It's not that hard, it just takes a lot of effort and will. And I think similarly with the mental health services. And you've got HIV and you've got depression, it will wear people out. And then you've got drugs that make people nasty and horrible, and weird and inconsolable. Then, it can wear a lot of people out. [GP9]

## Discussion

There are a number of limitations to this study which must be acknowledged. Firstly, the number of GP interviews conducted for the study is small. However, in keeping with the principles of qualitative epistemology, examining a small amount of qualitative data is a legitimate research tool, particularly if the objective of the analysis is – as in this case – to understand particular and contextual experiences rather than to make a claim to representativeness and generalisability [15,16]. In addition, although only sixteen interviews were conducted for this stage of the study, this sample can be seen to represent a large proportion of the general practice workforce currently providing care to gay men and HIV positive men in the three cities included in the study. For example, the eleven Sydney-based GPs interviewed represent more than one third of the total number of s100 HIV prescriber GPs working in the inner suburbs of Sydney. Secondly, although the issue of crystal meth use was not raised as an urgent problem by the GPs based in the other two sites – one a smaller Australian city, the other a rural-coastal city – this does not mean that crystal meth use or drug and alcohol use more broadly is not a problem at all in those locations. On the contrary, study data not reported here suggests that drug and alcohol use is a significant issue for many of the gay men and HIV positive men living in those cities.

Another important limitation to consider is that, in this study, the GPs often spoke about the two populations of gay men and HIV positive men as almost interchangeable. Sometimes a section of transcript that began with an observation on gay men would slip into a commentary on HIV – and vice versa – with little, if any, acknowledgement on the part of the GP that this was occurring. This may be because almost all of the HIV positive men these GPs treat are also gay, and that a very high proportion of the gay men they treat are also HIV positive. Each of these GPs is based in a practice that has adopted these populations as the focal point of their work, and which has over many years developed a reputation for expertise and understanding in the health and social issues that face those groups of men. The physical location of inner city Sydney is also important here, as all four practices are embedded within social and spatial networks associated with both gay and HIV positive 'community'. The fact that these GPs had little sense of the differences between these two groups – at least in terms of how they talked about issues such as crystal meth use – suggests that forcing an arbitrary distinction between them may not be useful, if only in this context.

Nonetheless there are some key implications that can be identified from the data reported in this paper. The experiences of these GPs suggests that health practitioners may benefit from broadening their understandings of how to anticipate and respond to problematic levels of crystal meth use in their patients. This is likely to increase the capacity of GPs and others health practitioners – particularly those working in gay men's health and HIV health promotion – to intervene at an early stage in order to mitigate the impact of crystal meth use on co-morbid mental illness and other health issues [7]. These GPs articulated a complex, multi-factorial model of the relationships between drug use, depression, sexuality and HIV in their patients. Thus, it is important for health practitioners to address each of these aspects in turn, rather than prioritising one as more urgent than another. Of course, there are some exceptions, such as the fact that the diagnosis and treatment of depression must usually wait until problematic levels of crystal meth use have been addressed. Equally, if drug use is affecting compliance with HIV medications then it is critical that drug use be firstly addressed in order to prevent the development of drug resistance. A 'stepped care' approach is likely to be most appropriate, particularly for managing co-morbid crystal meth use and depression [19]. Guidelines for the management of general practice patients with crystal meth use problems need to be further developed to address specific issues associated with gay men and HIV positive men [20].

Another implication relates to an understanding of the role that drug use plays in the lives of many gay men. Following Worth and Rawstorne, it is important to appreciate 'these phenomena as social relationships that are part of wider community norms and practices' [12]. GPs and other health practitioners must build trust with their gay male patients to ensure that patients are willing to disclose the full nature of their drug use and other potentially stigmatised practices. Evidence drawn from the quantitative arm of this study suggests that this group of GPs has a heightened awareness for detecting depression in gay men and HIV positive men [17], demonstrated in a high level of concordance between GP assessments and patient screening for depression [21]. In a similar sense, there is a clear need for education programs to train health practitioners – including referral specialists – to better appreciate the issues pertaining to crystal meth use in gay men and in HIV positive men. In addition to training, however, health services need to receive higher levels of resourcing and support to assist the health workforce to cope with the often difficult behaviour of patients experiencing problematic levels of crystal meth use. To avoid an ever increasing burden being placed on primary care and community services, there needs to be an increased resourcing of acute care and referral services to improve their availability, accessibility and expertise in crystal meth issues.

## Conclusion

This is the first study of its kind to report on the experiences of GPs who work with gay men and HIV positive men in Sydney in relation to crystal meth use and associated depression. Five key themes were identified. Commencing from the belief that crystal meth use is an increasing problem in a growing number of these men, these GPs also observed a strong if complex association between crystal meth use and depression. Patient crystal meth use is most often experienced by GPs as a series of challenges in the clinical management of depression, further complicated for those men who are HIV positive. These GPs also expressed serious concerns regarding the capacity of existing health services to cope with crystal meth use, including a perceived stigma among some health practitioners, and a consequent requirement for GPs to provide open-minded, non-judgemental care. Finally, particular issues facing the health workforce were identified including a sense of GPs being unprepared to cope with problematic levels of crystal meth use in their patients as well as a more general lack of capacity (and possible unwillingness) in the HIV and mental health workforce due to a growing range of contextual stressors. Despite some study limitations, key implications can be identified. Further investigation is required in order to consider whether these findings are reproducible in other general practice settings. Other issues that would be particularly useful to explore further include the relationship between depression, drug use and HIV medication regimens, as well as the characteristics and challenges facing the HIV general practice workforce in Australia.

## Abbreviations

GP: general practitioner; Crystal meth: Crystal methamphetamine

## Competing interests

The authors declare that they have no competing interests.

## Authors' contributions

DCS: contributed to the planning and reporting of the work described in the article. CEN: contributed to the conduct and reporting of the work described in the article. LM: contributed to the conduct and reporting of the work described in the article. SCK: contributed to the planning and reporting of the work described in the article. MRK: contributed to the planning and reporting of the work described in the article. All authors read and approved the final manuscript.

## Consent

Written informed consent was obtained from all participants to publish their data in deidentified form, including in peer reviewed journals. Deidentified written consent forms are available for review by the Editor-in-Chief of this journal.

## Pre-publication history

The pre-publication history for this paper can be accessed here:


